# Dissecting CAF Heterogeneity in Glioblastoma Reveals Prognostic Subtypes and a Central Regulatory Role for Spleen Tyrosine Kinase (SYK)

**DOI:** 10.3390/cancers17243942

**Published:** 2025-12-10

**Authors:** Ji-Yong Sung, Kihwan Hwang

**Affiliations:** Department of Neurosurgery, Seoul National University Bundang Hospital, Seoul National University College of Medicine, Seoul National University, Seongnam-si 13620, Republic of Korea

**Keywords:** glioblastoma, cancer-associated fibroblasts, tumor microenvironment, SYK, single-cell RNA-seq, prognosis, stromal heterogeneity, immunomodulation

## Abstract

**Background:** Cancer-associated fibroblasts (CAFs) are key components of the glioblastoma (GBM) microenvironment and contribute to tumor progression, immune evasion, and therapy resistance. However, their heterogeneity and clinical impact in GBM remain poorly defined. **Methods:** We performed an integrative transcriptomic analysis combining bulk and single-cell RNA sequencing (scRNA-seq) datasets to characterize CAF subtypes in GBM. Four CAF-associated transcriptional programs were defined based on canonical gene signatures: immune CAFs, myofibroblastic CAFs (myoCAFs), inflammatory CAFs (iCAFs), and antigen-presenting CAFs (apCAFs). Prognostic relevance was assessed using survival analyses, and hub genes were identified through network analysis. **Results:** CAF subtype-specific gene signatures were significantly associated with poor overall survival. Single-cell analysis revealed spatial heterogeneity of CAF activation, with immune and inflammatory CAF markers enriched in low-stemness tumor cells. SYK was identified as a central hub gene shared across CAF subtypes, suggesting its role in stromal signaling. **Conclusions:** Our study reveals CAF subtype-associated patterns with prognostic and functional relevance in GBM. Targeting CAF subpopulations and key mediators such as SYK may represent a promising therapeutic strategy in GBM.

## Simple Summary

Glioblastoma is an aggressive brain tumor with limited treatment options, and the tumor microenvironment plays a major role in its progression. Among stromal cells, cancer-associated fibroblasts (CAFs) are known to influence tumor growth and immune responses, but their diversity in glioblastoma has not been well understood. In this study, we analyzed both bulk and single-cell RNA sequencing data to identify CAF subtype-associated patterns within glioblastoma. We discovered four CAF populations—immune, inflammatory, antigen-presenting, and myofibroblastic—each associated with poor patient survival and unique immune and metabolic features. We also identified Spleen Tyrosine Kinase (SYK) as a key regulatory gene shared across these CAF subtypes. Our findings highlight the importance of stromal heterogeneity in glioblastoma and suggest that targeting CAF-related signaling, including SYK, may offer new therapeutic opportunities for this lethal disease.

## 1. Introduction

GBM is the most aggressive and lethal form of primary brain tumor in adults, characterized by rapid growth, extensive infiltration, and profound resistance to therapy. Although there have been improvements in surgery, radiation therapy, and chemotherapy, patients diagnosed with glioblastoma still face a poor prognosis, with median survival rarely surpassing 15 months. A growing body of research has shifted focus from tumor-intrinsic factors to the tumor microenvironment (TME), which plays a critical role in GBM progression, immune evasion, and therapeutic resistance. CAFs have been associated with immune-related processes and tumor-supportive phenotypes in various solid tumors, although the directionality of these interactions remains complex [1]. Traditionally associated with epithelial tumors, CAFs have only recently begun to attract attention in the context of GBM. However, their presence, heterogeneity, and functional roles in glioma remain largely undefined. Although CAFs have been described across a wide range of solid tumors, the existence and true cellular origins of fibroblast-like stromal populations in the brain remain a subject of debate. Recent studies suggest that CAF-like programs in GBM may arise not only from perivascular stromal cells but also through mesenchymal transition-related processes within tumor or immune cells. Considering this complexity, clarifying the potential cellular origins of CAF-like phenotypes is essential for interpreting stromal heterogeneity in GBM.

Recent studies in other solid tumors have revealed that CAFs are not a homogeneous population but rather consist of distinct subtypes with diverse biological roles [2]. These include immune-regulatory CAFs (immuneCAFs), contractile and matrix-remodeling myofibroblastic CAFs (myoCAFs), cytokine-secreting inflammatory CAFs (iCAFs), and antigen-presenting CAFs (apCAFs). Each subtype contributes differentially to tumor progression, immune modulation, and therapeutic response. Whether such functional diversity exists in GBM and how it might relate to prognosis or immune contexture is currently unknown [3,4].

While previous landmark studies have defined CAF subtypes across epithelial tumors, their relevance, transcriptional behavior, and clinical implications remain unclear in GBM. Therefore, our study specifically focuses on GBM to determine whether these established CAF programs are conserved, prognostically meaningful, and functionally distinct within the unique brain tumor microenvironment.

In this study, we comprehensively characterized CAF heterogeneity in GBM using both bulk RNA sequencing and single-cell RNA sequencing (scRNA-seq) datasets [5]. By applying well-defined subtype-specific gene signatures, we identified four CAF subtypes in the GBM microenvironment and evaluated their association with patient prognosis. Notably, high expression of these CAF signatures correlated with poor overall survival. At the single-cell level, we uncovered that CAF signature activity varied across different cell types, with immune- and inflammation-related CAF gene activity being higher in cells exhibiting lower stemness, suggesting a possible inverse relationship between cellular plasticity and immune interaction. Furthermore, through network-based analysis, we identified three hub genes that may coordinate CAF subtype function, among which Spleen Tyrosine Kinase (SYK) [6] emerged as a central regulator highly correlated with multiple CAF signatures. Through network-based analysis, we identified three central hub genes-SYK, JAK2, and TAF1. SYK encodes spleen tyrosine kinase, JAK2 encodes Janus kinase 2, and TAF1 encodes TATA-box binding protein-associated factor 1, all of which participate in key stromal or immunoregulatory signaling pathways [7,8,9,10].

SYK, a non-receptor tyrosine kinase known to mediate immune signaling and cellular adhesion, has not been previously implicated in CAF-mediated stromal regulation in GBM [11]. Our findings reveal the transcriptional heterogeneity of CAFs in GBM and highlight the prognostic significance of CAF subtype-associated patterns. By uncovering SYK as a key regulatory node [12], this study opens new avenues for targeting stromal components in GBM and provides a deeper understanding of the tumor–stroma–immune interface [5,13,14].

Table 1 summarizes the representative genes, biological characteristics, and functional implications of the four CAF-associated transcriptional programs identified in our study. These groups represent partially overlapping transcriptional patterns rather than discrete cellular populations

## 2. Methods

### 2.1. Bulk RNA-Seq Analysis

Bulk RNA sequencing (RNA-seq) data for glioblastoma (GBM) were obtained from The Cancer Genome Atlas (TCGA) through the Genomic Data Commons (GDC) Data Portal (https://portal.gdc.cancer.gov/projects/TCGA-GBM) (accessed on 1 May 2025). Raw HTSeq-count files (workflow type: “HTSeq-Counts”) and corresponding clinical metadata were downloaded in October 2024. All samples annotated as “Primary Solid Tumor” were retained for analysis. Clinical information, including overall survival (OS), progression-free interval (PFI), age, sex, and MGMT promoter methylation status, was matched to expression profiles using patient barcodes. RNA-seq count data were imported into R (v4.3.1) and processed using the DESeq2 package (v1.40.2). Genes with fewer than 10 counts across >80% of samples were removed. Normalization was performed using the DESeq2 median-of-ratios method, and variance-stabilizing transformation (VST) was applied for downstream correlation-, clustering-, and GSVA-based analyses. For visualization, VST-transformed expression values were z-scaled across samples.

Survival analysis was conducted using the GEPIA2 platform [15], which applies a Kaplan–Meier estimator and log-rank test thresholded by median expression cutoffs. To ensure reproducibility, we cross-validated GEPIA2 results using R packages survival (v3.5-7) and survminer (v0.4.9), employing Cox proportional hazards regression models with continuous signature scores. Functional enrichment analyses, including Gene Ontology (GO) Biological Process, KEGG pathways, Reactome pathways, and transcription factor networks, were performed using Metascape (https://metascape.org; accessed on 1 May 2025) [16] with the default parameters (minimum overlap = 3, *p*-value cutoff = 0.01, enrichment factor > 1.5). Enrichment clusters were visualized using Metascape’s network-based clustering and heatmap modules. Immune cell-type deconvolution was conducted using methodology from our prior publication, which integrates reference immune signatures across multiple platforms. Briefly, normalized expression values were processed using a consensus-based immune deconvolution algorithm that incorporates gene signatures from CIBERSORT, xCell, and MCP-counter, producing estimates for T-cell subsets, myeloid cells, NK cells, dendritic cells, and stromal populations. Protein–protein interaction (PPI) networks and transcriptional regulatory modules were constructed through ConsensusPathDB (CPDB) (v35). Interactions with confidence scores > 0.8 were retained. CPDB enrichment was performed using the “Induced Network Modules” tool with default parameters, allowing identification of intermediate hub genes linking CAF-associated signatures. Gene set variation analysis (GSVA) [17] was performed using the GSVA R package (v1.50.0) with a Gaussian kernel and non-parametric estimation. CAF signature scores (iCAF, immCAF, myoCAF, apCAF) were calculated using published gene sets, and scores were centered and scaled prior to statistical comparison. Associations between CAF scores, immune infiltration, and patient survival were analyzed using Spearman correlation and linear modeling (limma v3.56.2). All computational procedures were executed in R (v4.3.1) within a controlled reproducible environment, and complete parameter settings, gene lists, and analysis scripts are available upon reasonable request.

### 2.2. Single-Cell RNA-Seq Analysis

Single-cell transcriptomic datasets were obtained from GEO (GSE131928 and GSE84465). Raw sequencing matrices were downloaded as count matrices when available and preprocessed using the Seurat (v4.3.0) pipeline. Cells with >20% mitochondrial gene content, <200 detected genes, or >6000 detected genes (potential doublets) were excluded. After log-normalization (scale factor = 10,000), highly variable genes were identified using Seurat’s variance stabilization method (vst). Dimensionality reduction was performed using PCA, followed by UMAP embeddings. Cell clusters were generated using the Louvain algorithm at resolutions between 0.4 and 1.0, depending on dataset density. Cell-type annotations were assigned using canonical marker genes (e.g., PDGFRB/MCAM for vascular cells, MOG/OLIG1 for oligodendrocyte-like cells, GFAP/S100B for astrocyte-like cells) and cross-referenced with reference atlases using SingleR (https://bioconductor.org/packages/release/bioc/html/SingleR.html; accessed on 1 May 2025). CAF signature activity was quantified at the single-cell level using ssGSEA and confirmed with AddModuleScore in Seurat. Entropy-based stemness scores were computed using the StemID algorithm implemented in the RaceID framework [18]. Associations between stemness and CAF subtype activity were evaluated using Spearman correlation. Metabolic pathway activity was inferred using 83 KEGG metabolic signatures from MSigDB, applying ssGSEA across all malignant and stromal cell subsets. Differences in pathway activation were tested using Welch’s ANOVA followed by FDR correction.

### 2.3. Cancer Hallmark and Metabolic Pathway Analysis

Hallmark pathways were downloaded from the MSigDB (H collection). GSVA-derived enrichment scores were compared between CAF-high and CAF-low tumors using linear models adjusted for patient age, sex, and tumor purity.

Metabolic pathway profiling utilized seven major KEGG categories (lipid, carbohydrate, TCA cycle, amino acid, nucleotide, vitamin, energy metabolism). Pathway scores were normalized and centered before comparison. Telomere maintenance mechanisms (TMM), including ALT-related modules, were evaluated using previously published ALT gene sets.

## 3. Results

### 3.1. CAF Subtypes Predict Poor Prognosis and Shape Distinct Immune Microenvironments in GBM

To ensure consistency with previously established CAF biology, we applied canonical subtype signatures described in recent pan-cancer single-cell analyses. These validated CAF programs allowed us to systematically evaluate their prognostic and functional relevance specifically within the GBM microenvironment.

**Figure 1:**
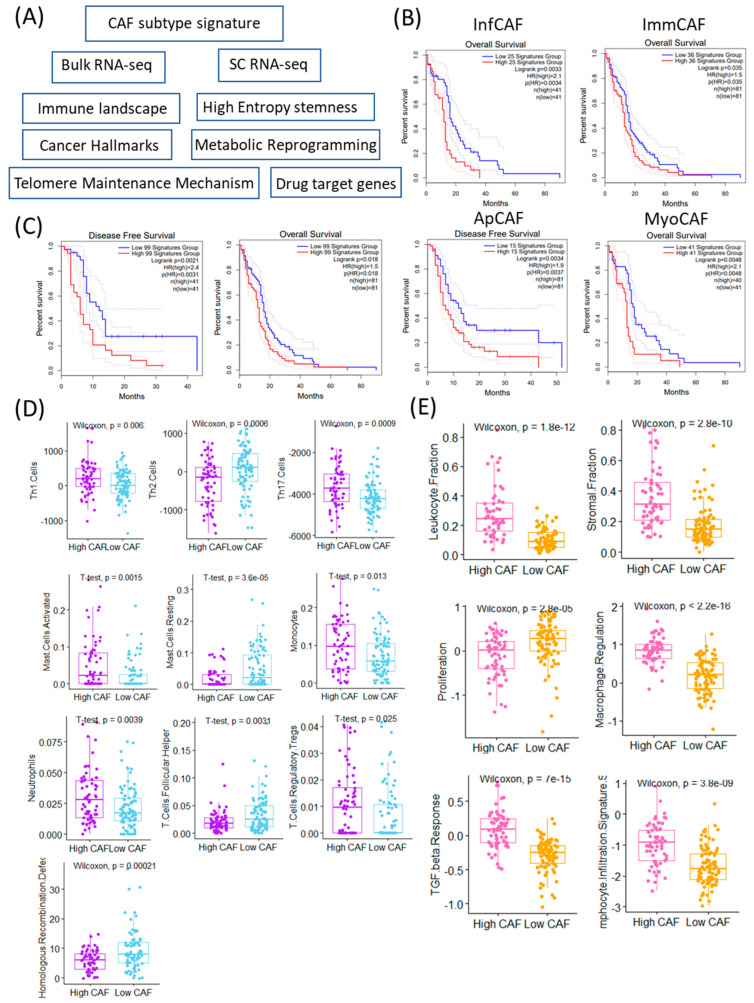
Clinical and immunological significance of CAF (Cancer-Associated Fibroblast) signature stratification. (**A**) Schematic overview of the study workflow identifying and analyzing CAF-related signatures in cancer samples. (**B**) Kaplan–Meier survival curves comparing overall survival (OS) and (**C**) disease-free survival (DFS) between patients stratified into high vs. low CAF signature groups across TCGA GBM. Log-rank *p*-values and sample sizes are indicated in each panel. (**D**) Boxplots showing significantly different immune cell infiltration (Th1, Th2, Th17 cells) between high and low CAF groups (Wilcoxon test). Differences in leukocyte and stromal fractions between high and low CAF groups, based on immune deconvolution (Wilcoxon test). Boxplots showing significant differences in immune cell subtypes (activated/resting mast cells and monocytes) based on CAF signature levels (*t*-test). Neutrophils, T-cell follicular helper, and regulatory T-cell proportions differ significantly between high and low CAF signature groups (*t*-test). (**E**) Functional signature scores (proliferation, macrophage regulation, TGF-beta response, lymphocyte infiltration) differ significantly between high and low CAF groups (Wilcoxon test). Homologous recombination defect scores are significantly higher in the high CAF group (Wilcoxon test).

To evaluate the clinical and immunological relevance of CAFs in GBM, we assessed the expression of CAF subtype-specific gene signatures using both bulk RNA-sequencing and single-cell RNA-sequencing datasets (Figure 1A). We focused on four previously defined CAF subtypes: inflammatory CAFs (infCAFs), immune-regulatory CAFs (immCAFs), antigen-presenting CAFs (apCAFs), and myofibroblastic CAFs (myoCAFs). Survival analysis using the TCGA-GBM cohort revealed that high expression of all four CAF subtype signatures was significantly associated with worse overall survival (infCAF: *p* = 0.0033; immCAF: *p* = 0.035; apCAF: *p* = 0.0034; myoCAF: *p* = 0.0048) (Figure 1B).

Importantly, the CAF–survival relationship reflects statistical significance rather than a major clinical effect, underscoring the need to distinguish between statistical and meaningful differences in survival.

Furthermore, the aggregate signature comprising all 99 CAF-related genes also showed a strong negative prognostic impact, with the high-expression group demonstrating significantly poorer survival (*p* = 0.0021) (Figure 1C). Patients were categorized into high and low CAF groups according to the mean expression levels of CAF-associated signature genes. Immune cell composition analysis revealed distinct immune landscapes between these two groups. The high CAF group exhibited significantly elevated activity of Th1 cells (*p* = 0.006), Th17 cells (*p* = 0.0009), mast cells (*p* = 0.0015), monocytes (*p* = 0.013), neutrophils (*p* = 0.0039), and regulatory T cells (Tregs, *p* = 0.025) (Figure 1D). In contrast, the low CAF group showed higher levels of Th2 cells (*p* = 0.0006), resting mast cells (*p* = 3.6 × 10^−5^), and follicular helper T cells (*p* = 0.0031). In addition to immune profiles, genomic and microenvironmental features differed significantly between CAF expression groups. Homologous recombination deficiency (HRD) scores were significantly elevated in the low CAF group (*p* = 0.00021), suggesting potential differences in genomic instability or DNA repair proficiency. Conversely, the high CAF group displayed significantly higher scores in leukocyte fraction (*p* = 1.8 × 10^−12^), stromal fraction (*p* = 2.8 × 10^−10^), macrophage regulation (*p* = 2.2 × 10^−16^), TGF-β response (*p* = 7 × 10^−15^), and lymphocyte infiltration signature (*p* = 3.8 × 10^−9^), all of which indicate a highly immunosuppressive and reactive stroma. Notably, the low CAF group was characterized by significantly higher activity of proliferation-related pathways (*p* = 2.8 × 10^−5^) (Figure 1E). These findings suggest that CAF abundance and subtype expression are not only associated with adverse prognosis but also reflect distinct immunological and stromal environments in GBM. The immunosuppressive and fibrotic features of the high CAF group may underlie therapeutic resistance, whereas the low CAF group may represent a more proliferative but genomically unstable tumor phenotype [19].

The subtype-specific CAF signatures used in this study were curated from previously validated CAF marker sets reported in single-cell and spatial transcriptomic studies across solid tumors. Representative markers for each subtype are summarized in Table 1.

### 3.2. CAF Signature Genes Are Associated with Cancer Hallmark Pathways and Metabolic Reprogramming in GBM

**Figure 2:**
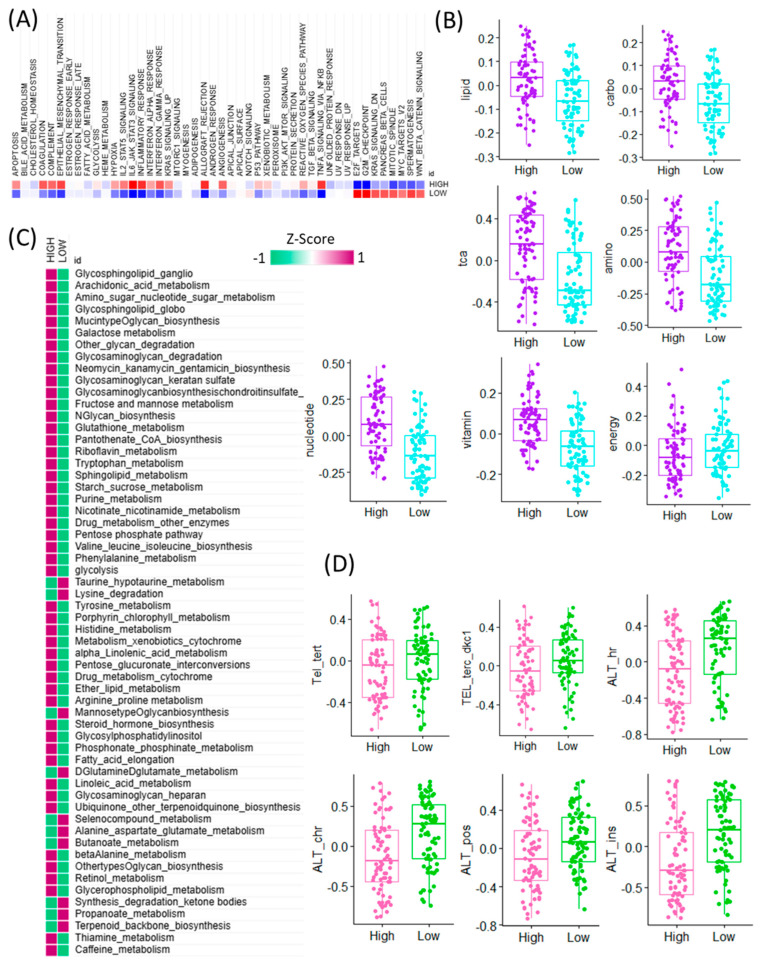
Transcriptomic comparison of CAF-high and CAF-low GBM tumors and their associations with cancer hallmarks, metabolic pathways, and telomere maintenance mechanisms. (**A**) Heatmap depicting the activity scores of canonical cancer hallmark pathways across TCGA GBM samples, stratified by high CAF and low CAF samples. Pathway activities were inferred using gene set enrichment analysis, and samples are ordered based on cancer hallmark pathway expression patterns. (**B**) Boxplots showing differential enrichment of major metabolic categories—lipid, carbohydrate (carbo), TCA cycle (TCA), amino acid (amino), nucleotide, vitamin, and energy metabolism—between CAF-high and CAF-low TCGA GBM tumors. (**C**) Heatmap showing the differential activity of KEGG-defined metabolic pathways between high-CAF and low-CAF GBM tumors. Pathway activity scores were calculated based on transcriptomic signatures, highlighting distinct metabolic programs associated with the degree of cancer-associated fibroblast (CAF) infiltration. Rows represent individual KEGG metabolic pathways, and columns represent GBM tumor samples stratified by CAF level. The color scale reflects relative pathway activity (e.g., enrichment z-scores), revealing a metabolic shift associated with CAF-high versus CAF-low microenvironments. (**D**) Boxplots presenting differential accessibility at key telomeric and ALT-associated chromatin regions, including Tel_tert, TEL_terc_dkc1, ALT_hr, ALT_chr, ALT_pos, and ALT_ins, between CAF-high and CAF-low groups.

To further explore the functional implications of CAF signature gene expression in GBM, we performed pathway-level analyses comparing high and low CAF expression groups using bulk RNA-seq data. Gene set enrichment analysis (GSEA) revealed that high CAF expression was predominantly associated with the activation of immune-related hallmark pathways, including inflammatory response, interferon gamma response, and TNFα signaling via NF-κB. In contrast, the low CAF group exhibited higher activity in proliferation-related and transcriptional regulation pathways such as E2F targets, G2M checkpoint, KRAS signaling down, pancreas beta cell signature, mitotic spindle, MYC targets, spermatogenesis, and WNT/β-catenin signaling (Figure 2A). Given the known interplay between CAFs and metabolic reprogramming, we next investigated the enrichment of metabolism-related pathways. Among seven major metabolic pathway categories analyzed, six showed significantly higher activity in the high CAF group, suggesting a strong association between CAF abundance and metabolic remodeling. Conversely, energy metabolism pathways were selectively upregulated in the low CAF group, indicating differential metabolic states across CAF subgroups (Figure 2B). To obtain a more granular understanding, we conducted enrichment analysis across 83 metabolic pathways curated in the KEGG database (Figure 2C). This analysis revealed that the majority of metabolic pathways were more active in the high CAF group, particularly those involved in taurine and hypotaurine metabolism, lysine degradation, mannose-type O-glycan biosynthesis, D-glutamine and D-glutamate metabolism, selenocompound metabolism, alanine, aspartate and glutamate metabolism, and butanoate metabolism. These findings suggest that GBM samples with high CAF signature gene expression exhibit broad activation of amino acid and glycan-related metabolic programs. In addition, we evaluated the potential relationship between CAF signature expression and telomere maintenance mechanisms (TMM) (Figure 2D). Notably, components of the alternative lengthening of telomeres (ALT) pathway were significantly enriched in the low CAF group, indicating an inverse association between CAF abundance and telomere instability. This result suggests a link between stromal activation and genomic maintenance programs in GBM. Collectively, these findings highlight that CAF signature gene expression in GBM is tightly connected to immune activation, metabolic reprogramming, and telomere biology, providing deeper insights into the complex roles CAFs play in shaping tumor behavior [20].

### 3.3. CAF Signature Activity Varies by Cell Type and Stemness at the Single-Cell Level

**Figure 3:**
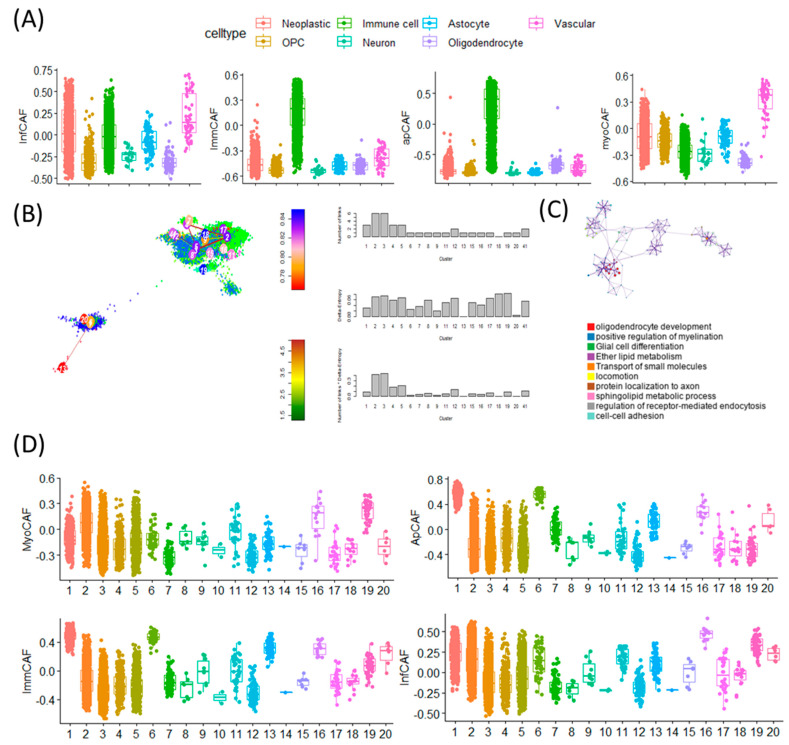
Single-cell transcriptomic analysis reveals distinct CAF subtype activities, stemness gradients, and pathway enrichments across GBM cellular states. (**A**) Boxplots showing abundance scores of four CAF subtypes—inflammatory CAF (iCAF), immunomodulatory CAF (imCAF), antigen-presenting CAF (apCAF), and myofibroblastic CAF (myCAF)—across seven major GBM single-cell types. Each color represents a distinct cell population: Neoplastic (red), OPC-like (yellow), Immune cells (green), Neuron-like (cyan), Astrocytes (pink), Oligodendrocytes (purple), Vascular cells (magenta) These plots illustrate the differential activation of CAF subtype transcriptional programs across diverse cellular compartments of the GBM microenvironment. (**B**) t-SNE map of GBM single cells generated using the StemID algorithm. Cells are colored by StemID-defined cluster number, with clusters labeled directly on the plot. Arrows indicate inferred lineage trajectories. StemID derives stemness based on transcriptional entropy, shown on the accompanying entropy scale bar: higher entropy = higher transcriptional plasticity and stem-like potential. Bar plots to the right summarize entropy values across the 20 clusters. (**C**) Biological pathways enriched in high-entropy clusters-representing transcriptionally plastic, stem-like glioblastoma cell states. The pathway enrichment map and network highlight dominant programs characteristic of these clusters, including Oligodendrocyte development, Positive regulation of myelination, Glial cell differentiation, Ether lipid metabolism, Transport of small molecules, Locomotion and migration, Protein localization to axon, Sphingolipid metabolic process, Regulation of receptor-mediated endocytosis, Cell–cell adhesion. These pathways collectively demonstrate that high-entropy clusters engage developmental, metabolic, and invasive programs, consistent with GBM stem-like phenotypes. (**D**) Boxplots showing the distribution of CAF subtype scores (myCAF, apCAF, imCAF, iCAF) across the 20 StemID transcriptional clusters. Distinct score patterns across clusters highlight significant heterogeneity in CAF program activation among GBM cellular states.

To investigate the cellular specificity of CAF signature gene activity, we performed single-cell level analysis across different cell types within the GBM tumor microenvironment. Using CAF subtype-specific gene sets, we observed distinct activation patterns corresponding to specific cellular lineages. Notably, inflammatory CAF (infCAF) and myofibroblastic CAF (myoCAF) signatures showed the highest expression in vascular cell populations, while immune-regulatory CAF (immCAF) and antigen-presenting CAF (apCAF) signatures were predominantly enriched in immune cells (Figure 3A). These findings suggest that CAF-related programs are variably active across the tumor stroma and immune compartments. To explore how cell-intrinsic plasticity influences CAF signature expression, we next examined single-cell entropy scores-a proxy for cellular stemness-across multiple GBM scRNA-seq datasets (Figure 3B). Stemness in malignant GBM cells was estimated using StemID-derived transcriptional entropy scores, where high entropy represents a more stem-like transcriptional program. These entropy-based stemness scores were then compared with CAF subtype signature activity at the single-cell level.

In this analysis, lower transcriptional entropy was interpreted as an indicator of lower stemness, and the term ‘low-entropy’ has therefore been replaced with the more intuitive descriptor ‘cells with lower stemness’ throughout the manuscript.

Cells with high entropy, indicative of greater stem-like potential, were enriched for gene programs related to oligodendrocyte development, positive regulation of myelination, and glial cell differentiation (Figure 3C). These stem-like populations displayed elevated activity of the myoCAF signature, suggesting a link between mesenchymal traits and cellular plasticity. In contrast, low-entropy cells exhibited higher expression of the immCAF signature, which is typically associated with immune modulation. Together, these results indicate that CAF signature gene activity is not uniformly distributed across all tumor-associated cell types but rather is shaped by both cell identity and stemness state (Figure 3D). This highlights a dynamic interaction between stromal signaling and tumor cell plasticity, offering insights into how CAF programs may contribute to GBM heterogeneity and therapy resistance [21].

### 3.4. Network Analysis Identifies SYK as a Central Mediator of CAF-Associated Gene Signatures in GBM

**Figure 4:**
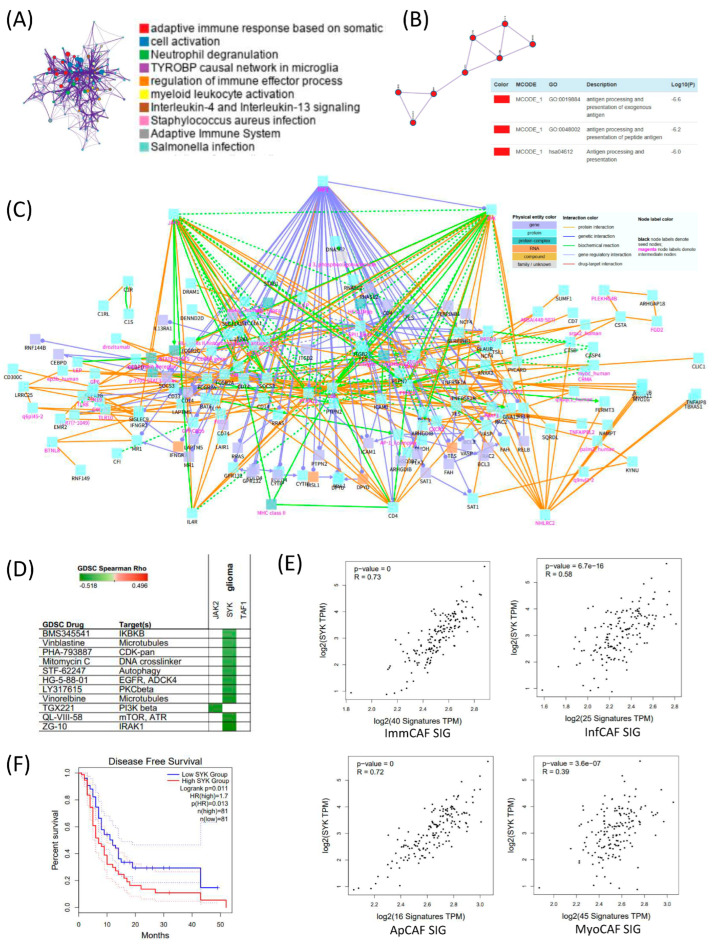
Integrated analysis of CAF-associated immune regulation and SYK-mediated prognostic significance in TCGA GBM. (**A**) Differentially expressed genes (DEGs) between high and low cancer-associated fibroblast (CAF) groups in TCGA GBM dataset were identified and subjected to pathway enrichment analysis. Prominent immune-related pathways are highlighted. (**B**) Protein–protein interaction (PPI) network of DEGs constructed using MCODE clustering, with major clusters annotated by Gene Ontology (GO) terms related to antigen processing and presentation. (**C**) Functional network of the high-CAF group constructed using ConsensusPathDB (CPDB). Genes and pathways are visualized with physical entity and interaction types annotated. Notably, immune cell signaling, cytokine response, and antigen presentation modules are enriched. (**D**) Drug response correlation analysis using Genomics of Drug Sensitivity in Cancer (GDSC) dataset. SYK expression shows significant correlation with sensitivity to several targeted agents, particularly those affecting microtubules, DNA replication, and kinase signaling. (**E**) Correlation analysis between SYK expression and four CAF subtype signature gene sets. SYK expression is significantly positively correlated with all CAF subtype signatures, indicating its potential regulatory role. (**F**) Kaplan–Meier disease-free survival analysis based on SYK expression levels. Patients with high SYK expression (red) exhibit significantly shorter survival compared to those with low SYK expression (blue), highlighting its prognostic relevance in GBM.

To explore the regulatory framework underlying CAF signature gene expression in GBM, we conducted differential gene expression analysis between high CAF and low CAF groups in bulk RNA-seq data, followed by functional annotation. Gene Ontology (GO) enrichment analysis of upregulated genes in the high CAF group revealed significant enrichment in pathways related to adaptive immune response, cell activation, neutrophil degranulation, and the TYROBP causal network in microglia, suggesting immunologically active and potentially pro-inflammatory stromal environments (Figure 4A). Subsequent protein–protein interaction (PPI) network analysis showed that these differentially expressed genes were enriched in antigen processing and presentation of exogenous antigens and peptide antigens, reinforcing the immunomodulatory role of the CAF-high microenvironment (Figure 4B). To identify potential regulatory mediators, we constructed a gene network of the CAF signature using the ConsensusPathDB (CPDB) interaction database. Network topology analysis identified JAK2, SYK (Spleen Tyrosine Kinase), and TAF1 as key intermediate hub genes connecting CAF subtype-specific gene signatures (Figure 4C). Figure 4C depicts the interaction network linking CAF subtype–associated genes and demonstrates that signaling pathways from multiple CAF signatures converge onto several intermediate hub genes. Notably, SYK emerges as the most interconnected stromal hub, suggesting a shared regulatory architecture across CAF subtypes.

To assess the therapeutic relevance of these hub genes, we queried the Genomics of Drug Sensitivity in Cancer (GDSC) database and found that their expression was associated with sensitivity to compounds targeting IRAK1, mTOR, and ATR, highlighting possible druggable vulnerabilities linked to CAF activity (Figure 4D). Among the identified hub genes, SYK showed the highest correlation with CAF subtype gene sets, particularly with immCAF and apCAF signatures (Figure 4E). This strong association suggests that SYK may play a central regulatory role in orchestrating immune-modulatory CAF phenotypes in GBM. In Figure 4F, the median disease-free survival of the high-SYK group was approximately 4–5 months shorter than that of the low-SYK group. Although this difference reached statistical significance (log-rank *p* = 0.011; HR = 1.7), its clinical significance should be interpreted with caution.

While the survival difference of approximately 4–5 months is notable, we distinguish statistical significance from clinical relevance, and refrain from overinterpreting this difference without validation in independent cohorts.

Together, these findings position SYK not only as a key mediator of CAF-associated signaling but also as a candidate biomarker and therapeutic target in GBM stromal biology [22].

## 4. Discussion

In this study, we comprehensively dissected the heterogeneity of cancer-associated fibroblasts (CAFs) in GBM and elucidated their biological, immunological, and prognostic relevance using both bulk and single-cell transcriptomic approaches. While CAFs have been extensively characterized in various solid tumors, their roles in GBM remain relatively unexplored. Our findings reveal that CAFs are not only present but exhibit functionally distinct subtypes within the GBM tumor microenvironment (TME), including inflammatory CAFs (infCAFs), immune-regulatory CAFs (immCAFs), antigen-presenting CAFs (apCAFs), and myofibroblastic CAFs (myoCAFs). Each subtype shows unique expression patterns and associations with clinical outcome, immune landscape, and metabolic rewiring, underscoring the complexity and relevance of stromal components in GBM [23,24].

Notably, high expression of all four CAF subtype signatures was associated with significantly worse overall survival in TCGA GBM patients, suggesting that CAF activation broadly contributes to tumor aggressiveness. The immune profiles of high CAF tumors were enriched for Th1, Th17, neutrophils, monocytes, and regulatory T cells, indicating an immunosuppressive and inflammatory milieu. It is important to note that the associations observed between CAF subtype signatures and lymphocyte infiltration patterns do not imply unidirectional causality. Alternative possibilities—including lymphocytes modulating CAF phenotypes or GBM tumor cells coordinately influencing both stromal and immune compartments—remain equally plausible. Our findings should therefore be interpreted as correlative rather than indicative of a primary regulatory direction.

In contrast, low CAF tumors displayed features such as Th2 and follicular helper T cell enrichment and increased proliferation, potentially reflecting a more proliferative but less immune-reactive state. These findings demonstrate that CAF expression stratifies GBM patients into biologically distinct subgroups with divergent immune and stromal profiles. These correlations should not be interpreted as evidence that CAFs directly regulate lymphocyte infiltration; rather, they reflect stromal–immune interactions that could be shaped by multiple cellular sources within the GBM microenvironment.

At the metabolic level, high CAF tumors were enriched in multiple metabolic pathways, particularly those involving amino acid metabolism and glycan biosynthesis, while energy metabolism was more prominent in low CAF tumors. These distinct metabolic profiles suggest that CAFs may reshape the tumor’s metabolic landscape in a manner that supports immune modulation and stromal remodeling. Furthermore, we observed that alternative lengthening of telomeres (ALT) pathways were significantly enriched in the low CAF group, linking telomere maintenance mechanisms with CAF activity and hinting at potential interactions between stromal composition and genomic stability. Single-cell RNA-seq analysis further demonstrated that CAF signature gene activity varies across cell types and is influenced by stemness. We observed that myoCAF signatures were elevated in vascular and high-entropy (stem-like) cell populations [25], while immCAF signatures were enriched in low-entropy (more differentiated) immune cell subsets. These data suggest that CAF programs are dynamically regulated in response to the developmental state of tumor and stromal cells, and that certain CAF subtypes may preferentially interact with or arise from specific cell lineages.

Importantly, our network analysis identified SYK (Spleen Tyrosine Kinase) as a central hub gene connecting multiple CAF subtype signatures, particularly immCAFs and apCAFs [2]. SYK is a non-receptor tyrosine kinase with established roles in immune signaling, but its role in CAF biology has not been previously highlighted in GBM [6]. Its strong correlation with multiple CAF signatures, poor prognosis, and association with drug sensitivity to mTOR, ATR, and IRAK1 inhibitors positions SYK as a promising therapeutic target and biomarker within the GBM stroma. While CAF-targeted therapies have been proposed in other cancer types, our results suggest that SYK inhibition could disrupt pro-tumorigenic CAF signaling pathways in GBM [1]. Importantly, several clinically approved agents, including fostamatinib and dasatinib, possess established SYK inhibitory activity. Given that our network analysis identifies SYK as a central stromal hub across multiple CAF subtypes, these SYK inhibitors may have the potential to modulate CAF-associated signaling programs within the GBM microenvironment. Although direct evidence in GBM-derived CAFs is still limited, the availability of these agents provides a strong mechanistic rationale for future preclinical studies targeting the CAF–SYK axis [26].

Given the exploratory nature of the drug–response correlations derived from the GDSC database, these results should be interpreted with caution, particularly in the context of GBM and the limited generalizability of pan-cancer pharmacogenomic resources. While SYK expression showed associations with sensitivity to several targeted agents, such correlations are inherently dependent on heterogeneous cancer cell lines and may not directly reflect the biology of the GBM microenvironment. Accordingly, these findings are presented as hypothesis-generating rather than conclusive, underscoring the need for future functional validation. In parallel, the stratification of SYK expression into high and low groups was determined using median-based thresholds with survival separation cross-validated across datasets, providing a statistically transparent framework for interpreting the prognostic implications of SYK activity.

Although our computational and network-based analyses strongly support SYK as a central regulatory hub across multiple CAF subtypes, we acknowledge that direct experimental validation of SYK-mediated effects was beyond the scope of the present study. To address this limitation, future work will include in vitro and in vivo perturbation assays to evaluate the causal role of SYK in CAF-associated signaling and to determine whether targeting SYK can modulate stromal–immune interactions in GBM.

Collectively, our study presents a multi-layered view of CAF biology in glioblastoma, linking stromal heterogeneity to immune contexture, metabolic reprogramming, and telomere maintenance. These findings broaden our understanding of the GBM microenvironment and propose CAF subtypes and SYK as key components that could be exploited for biomarker development and targeted intervention. Future studies should aim to validate these findings in spatial and functional contexts, and explore the efficacy of combining CAF-modulatory strategies with existing immunotherapies or metabolic interventions in GBM [27,28].

The existence, origin, and biological significance of CAF-like populations in glioblastoma remain topics of ongoing debate, largely due to the unique nature of the CNS microenvironment and the relatively small number of stromal cells detected in GBM. In recognition of these complexities, we have interpreted our findings with caution and expanded the discussion to outline current concepts regarding the putative cellular origins of GBM-associated CAFs, including perivascular stromal populations and mesenchymal transition-related states. Furthermore, because the interpretation of multi-parametric transcriptomic analyses carries an inherent risk of chance associations, we have deliberately avoided overstatement and have framed our conclusions within the statistical limitations of the dataset. We also acknowledge the strengths and constraints of the foundational datasets used in this study and emphasize that future experimental and spatially resolved studies will be essential to validate and refine the CAF-related mechanisms proposed here.

## 5. Conclusions

Our study reveals the presence of functionally CAF subtype-associated patterns in GBM and underscores their critical roles in shaping the tumor microenvironment through immune modulation, metabolic remodeling, and telomere regulation. The identification of SYK as a central hub gene across CAF programs highlights its potential as a novel stromal therapeutic target. CAF subtypes highlight distinct associations with the GBM microenvironment, reflecting the complex and multidirectional nature of stromal–immune–tumor interactions. These findings offer valuable insights into GBM stromal biology and provide a foundation for CAF-targeted therapeutic strategies.

## Figures and Tables

**Table 1:** Summary of the four CAF signature groups identified in glioblastoma.

CAF Signature Group	Representative Genes	Key Biological Attributes	Functional/Immunological Features	Notes
**Immune CAF (immCAF)**	*CXCL12*, *CCL2*, *CSF1*, *IL6*	Immune-related signaling; chemokine and cytokine secretion	Associated with recruitment of monocytes, Tregs, and Th17 cells; linked to immunosuppressive microenvironment	Highly correlated with SYK signaling activity
**Inflammatory CAF (iCAF)**	*IL1B*, *TNF*, *CXCL1*, *CXCL8*	Inflammatory cytokine production; NF-κB-driven pathways	Promotes inflammatory remodeling; associated with high stromal fraction and TGF-β response	Often overlaps with immune CAF programs
**Antigen-presenting CAF (apCAF)**	*HLA-DRA*, *HLA-DRB1*, *CD74*	MHC-II-related antigen presentation machinery	May modulate T cell activation/exhaustion; enriched in immune-interactive niches	Shares transcriptional convergence with SYK and JAK2 hubs
**Myofibroblastic CAF (myoCAF)**	*ACTA2*, *TAGLN*, *COL1A1*, *PDGFRB*	Extracellular matrix remodeling; contractile phenotype	Linked to fibrotic stroma, macrophage regulation, and stromal activation	More enriched in high-stemness/high-entropy GBM cell contexts

## Data Availability

The original data presented in this study are included within the article. Further inquiries can be directed to the corresponding authors.
